# Integrative ecological and molecular analysis indicate high diversity and strict elevational separation of canopy beetles in tropical mountain forests

**DOI:** 10.1038/s41598-020-73519-w

**Published:** 2020-10-07

**Authors:** Andreas Floren, Thomas von Rintelen, Paul D. N. Hebert, Bruno Cancian de Araujo, Stefan Schmidt, Michael Balke, Raden Pramesa Narakusumo, Djunijanti Peggie, Rosichon Ubaidillah, Kristina von Rintelen, Tobias Müller

**Affiliations:** 1 Department of Animal Ecology and Tropical Biology, Biocenter, University of Würzburg, Hans-Martin-Weg 5, 97074 Würzburg, Germany; 2 Bavarian State Collection of Zoology, Münchhausenstr. 21, 81247 Munich, Germany; 3 Museum für Naturkunde-Leibniz Institute for Evolution and Biodiversity Science, Invalidenstraße 43, 10115 Berlin, Germany; 4 Centre for Biodiversity Genomics, University of Guelph, Guelph, ON N1G 2W1 Canada; 5 Zoology Division (Museum Zoologicum Bogoriense), Research Center for Biology, Indonesian Institute of Sciences, Jl. Raya Jakarta-Bogor KM 46, Cibinong, Bogor 16911 Indonesia; 6 Museum of Natural History Karlsruhe, Erbprinzenstr. 13, 76133 Karlsruhe, Germany; 7 Department of Bioinformatics, Biocenter, University of Würzburg, Am Hubland, 97074 Würzburg, Germany

**Keywords:** Ecosystem ecology, Ecosystem ecology

## Abstract

Tropical mountain forests contribute disproportionately to terrestrial biodiversity but little is known about insect diversity in the canopy and how it is distributed between tree species. We sampled tree-specific arthropod communities from 28 trees by canopy fogging and analysed beetle communities which were first morphotyped and then identified by their DNA barcodes. Our results show that communities from forests at 1100 and 1700 m a.s.l. are almost completely distinct. Diversity was much lower in the upper forest while community structure changed from many rare, less abundant species to communities with a pronounced dominance structure. We also found significantly higher beta-diversity between trees at the lower than higher elevation forest where community similarity was high. Comparisons on tree species found at both elevations reinforced these results. There was little species overlap between sites indicating limited elevational ranges. Furthermore, we exploited the advantage of DNA barcodes to patterns of haplotype diversity in some of the commoner species. Our results support the advantage of fogging and DNA barcodes for community studies and underline the need for comprehensive research aimed at the preservation of these last remaining pristine forests.

## Introduction

Biodiversity is a basic metric of ecosystems. Yet for tropical forests, arguably the most diverse habitats on earth, knowledge about the magnitude of species richness and the composition of diversity is very incomplete^1,2^. This applies in particular to tropical mountains which account for a considerable proportion of total biodiversity^3^. Although it is well known that trees harbour a rich arthropod fauna^4–6^ which perhaps make the largest contribution to overall diversity, there is still far too little data to assess the importance of arboreal diversity for ecosystem processes and functions. Comprehensive research, even from the largest rainforest areas on earth is incomplete although such studies are urgently needed to investigate the distribution of biodiversity and the impacts of anthropogenic disturbance^1,7^. Only a few studies have tried to examine biodiversity for all strata in a tropical rainforest comprehensively, such as on Sulawesi^8^ or in the IBISCA project (Investigating the Biodiversity of the Soil and Canopy Arthropods)^4^. The value of such surveys lies in the establishment of a sound database which can be used to examine whether less comprehensive studies are consistent with the findings of such large-scale monitoring programs.

Essential for assessing and understanding the extent of overall biodiversity is the analysis of beta-diversity which measures the compositional similarity between habitats^9,10^. Mountain forests are particularly interesting in this context because the biotic and abiotic conditions change at short distance forcing species to adopt to the differing conditions^11^. Although elevational gradients should affect the diversity and composition of the canopy communities, the extent of these effects is poorly known. The few studies that have examined tree communities report high beta diversity between elevational forests without more detailed results^12^.

Although the importance of canopy research was not pursued for a long time because of the difficult access, several methods now allow the study of arboreal biodiversity. Among these, insecticidal knock down (fogging) provides the most comprehensive information on the diversity and composition of arboreal communities^13^. But while field work can be carried out quickly and without complications, the processing of large bulk samples requires many years of taxonomic effort to assemble information on species composition^4^. This bottleneck can be overcome by applying high-throughput genetic methods (DNA barcoding) which can characterize hyper-diverse taxa like Coleoptera with great accuracy and in a comparatively short time^14–16^. This is particularly important because most tropical taxa are poorly taxonomically known and most species await formal description^2,6^. The comparison of molecular sequences also makes it possible to study how species change between forests with high accuracy.

In this study we investigate the similarity of arthropod communities found on individual trees in a sub-montane and a montane tropical forest. Fogging provides a comprehensive sample of the arthropod fauna allowing community-level analysis^17^. Here we test the extent to which differences in habitat conditions between two elevational forests lead to differences in the arboreal arthropod communities. Besides comparing major taxa composition, we performed an in-depth analysis of canopy beetles which were identified by morphotypes and DNA barcodes. Using DNA barcodes improves and expands the analysis considerably by facilitating the delineation of cryptic species^18–20^ and by making it possible to examine genetic differences between species (see below). Based on these results, we ask how strongly beetle communities differ in structure, alpha and beta-diversity within and between sites. Theory predicts that species in tropical forests should possess narrow thermal tolerances which limit their distribution resulting in the occupation of discrete elevational ranges^20,21^, leading to spatial separation of populations, reduced gene flow, and potentially to allopatric speciation^21,22^. Until now, little is known about how species arise and persist on tropical mountain^3^. In order to gain further knowledge on this subject, we use sequence differences to analyse evolutionary divergence among beetle populations from the sub-montane and the montane forests to test the hypothesis that differences between sites have resulted in genetic differences.

This research was carried out on two pristine forest sites within Mount Halimun-Salak National Park on Java, Indonesia. These forests are among the most diverse on earth^23^ but have rarely been subjected to extensive biodiversity study. As in most tropical countries, these forests are exposed to the ongoing threat of transformation into plantations^24^. In fact, in the Halimun mountains no forests are left below 1000 m altitude.

## Material and methods

### Sample sites

Forests were studied at two sites in the Mount Halimun-Salak National Park, Cikaniki at 1100 m and Gunung Botol (Botol) at 1700 m altitude a.s.l. which were separated by an aerial distance of six kilometres. From an elevation of 1000 m, the mountain is fully covered by forests. The park area comprises 113,357 ha of which several areas are regenerating following heavy deforestation^25^. Natural forest types can be divided into lowland rain forest (100–1000 m) dominated by the colline zone (500–1000 m), sub-montane (1000–1500 m), and montane forest (1500–1929 m). To our knowledge no comprehensive study had been performed in the Halimun mountain forest to assess species diversity. According to Halimun-Salak National Park reports, more than 700 species of flowering plants, belonging to 390 genera and 119 families occur in the park^26^. In total, 28 trees were selected for analysis at an average distance of 200 m with many other trees and vegetation growing between them. All field work was performed during the wet season in April 2016. Only tree species were chosen that were typical for the respective altitude. Several specimens of two tree species, *Lithocarpus indutus* (Fagaceae) and *Sloanea sigun* (Elaeocarpaceae), were fogged in both forests to permit direct comparison of insect communities on these tree species at both elevations (S-Tab. 1). Only trees were sampled whose crowns were not too close to other trees and which had a low load of epiphytes and lianas to exclude arthropods from other plants. All tree-specific parameters (girth at breast height, leaf cover, tree height, and size of the collecting sheet, S-Tab. 2) did not differ between sites (Wilcox-test, n.s.). Tree-family composition was weakly significantly associated with study site (Fisher’s exact-test p = 0.02).

### Arthropod sampling

Arthropods were collected quantitatively by insecticidal knock-down (fogging) and in a tree-specific way^27^. Natural pyrethrum was used as an insecticide because it quickly degrades in sunlight, ensuring no persistent damage. Immediately after contact with the insecticidal fog, insects dropped into collecting sheets installed beneath each tree crown from which they were transferred into vials filled with absolute ethanol. Fogging was done in the early morning or early evening when there was no wind and the fog could reach the treetops. A drop time of 90 min was allowed before the catch of arthropods was harvested. Only characteristic trees for each forest were sampled. They were not shaded by higher canopy; they did not overlap with neighbouring trees, and they did not have a large epiphyte load to avoid sampling arthropods from other plants. All major arthropod groups were separated and compared between trees after standardisation of specimen numbers on a per square meter collection area. A detailed analysis of species diversity was performed using DNA barcodes for the Coleoptera. Their large contribution to biodiversity and their specialisation on host plants, which should result in high predictability of associated species, explain their frequent use in ecological analyses^11,28^.

### Specimen selection and DNA sequencing

All beetle specimens were barcoded. Before DNA sequencing, specimens were sorted by size. A single leg was removed from larger specimens while DNA was extracted from the entire body of smaller specimens using a non-destructive process. The specimens were analysed at the Canadian Centre for DNA Barcoding (CCDB). DNA extraction, PCR amplification, and Sanger sequencing were conducted using standardized high-throughput protocols^29,30^ (https://www.ccdb.ca/resources.php). The target region is a 658 bp region of the mitochondrial cytochrome *c* oxidase (COI) often referred to as COI-5P^31^. The sequences were aligned using the BOLD aligner, and divergence values were calculated using the Kimura 2-parameter model (K2P)^32^. Each sequence that met minimum quality requirements was assigned a Barcode Index Number (BIN) by BOLD. A BIN is a globally unique identifier assigned to a cluster of sequences that has been shown to closely correspond to a biological species^14^. BINs provide molecular operational taxonomic units (MOTUs), an interim taxonomic system to delineate genetic units prior to detailed taxonomic studies including morphology^33^. BINs and species are used interchangeably throughout this paper. The BIN algorithm was designed to provide a conservative estimate of the actual species count. It differs in this regard from some other widely used approaches for delineating MOTUs from barcode data, such as GMYC (General Mixed Yule Coalescent), a result shown in many studies. The correspondence between BIN counts and species counts is very high in beetles as noted by Pentinsaari and collaborators^34^ in their barcoding study on the Finnish beetle fauna. There was 92% concordance between recognized beetle species and taxa delineated by BINs. Cases of discordance often involved overlooked cryptic species.

### Barcode of Life Data System (BOLD) management

Prior to sequence analysis, each specimen was assigned a unique ID in the INFOC (IndoBioSys Fogging Coleoptera) project inside the IndoBioSys (Indonesian Biodiversity Discovery and Information System) campaign on BOLD. Each specimen record in BOLD is accompanied by an image of the voucher specimen and information on its collector, collection date, locality, geographic coordinates, altitude, voucher depository, and barcode sequence. In addition, each record includes a detailed Laboratory Information Management System (LIMS) report, primer information, and trace files. All records are publicly available through the BOLD system (https://www.boldsystems.org) through the following dx.doi.org/10.5883/DS-INFOGCOL and the sequences are also available through GenBank accession numbers MK080571-MK084473.

### Characterisation of sequences

**Figure 1:**
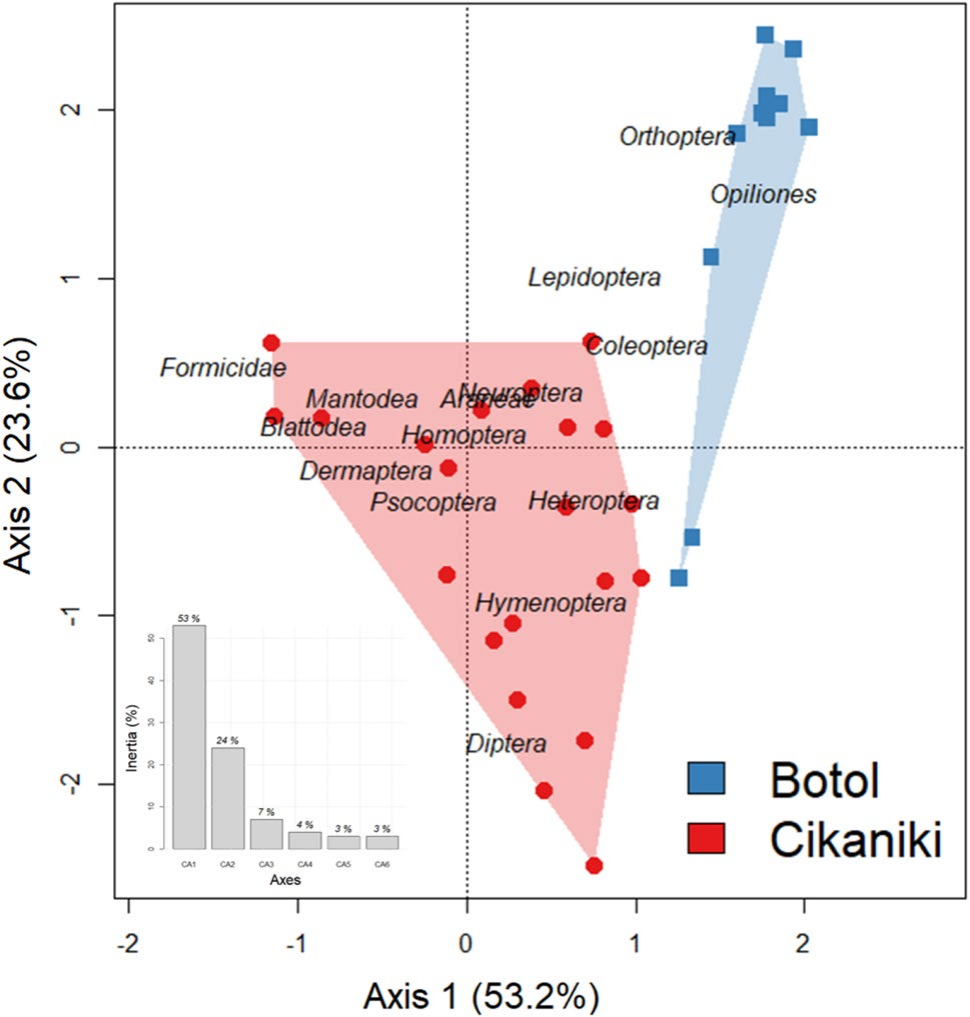
Correspondence analysis of the major arthropod groups collected from all fogged trees (coloured symbols) clearly separate the high elevation forest (blue polygon) from the lower forest (red polygon). The first two axes explain 76.8% of the variation. The extremely abundant Formicidae on axis 1 are strongly associated with the lower elevational forest while the two forest types are separated on both axes. The inlay figure shows the percentage of the total inertia explained by each of the first six axes.

Sequencing success was roughly 50% for all beetles. Mean sequencing success between trees at both sites showed no significant difference (Botol and Cikaniki, Wilcox test p = 0.09). We downloaded all specimen records for Coleoptera with COI sequence data in BOLD (1/9/2018). From these 217,815 sequences, we selected 145,198 sequences with a length of 500–700 nucleotides. These records included representatives of 32,705 unique BINs. These data were used to construct a local BLAST database using the NCBI BLAST software to compare the collected beetle sequences with those in BOLD. The alignment of sequences from all fogged beetles was generated by BOLD. Start and end regions of this multiple alignment were trimmed (S-Fig. 1) resulting in a multiple sequence alignment of 571 bp for 3668 sequences. The pairwise genetic distances were calculated by the Kimura two-parameter model (K2P) as implemented in the R-package APE^35^. In order to contrast the distribution of genetic distances between beetles in BOLD with our fogging data, we randomly selected 3668 sequences from the total Coleoptera sequence data and calculated their K2P distances against all sequence alignments (6.7 million local alignments). Only local alignments with a length greater than 200 bp (19.7%) were used in these analyses. Alignments were calculated based on the Biostrings R package^36^.

### Statistics

Statistical analyses were performed in R version 3.4.3^37^. We used the vegan package^38^ to conduct correspondence analysis for the major taxa composition of canopy communities. A scree-plot was added to visualise the explained variance of the major axes. The iNEXT^39^ package was used to perform rarefaction analysis with extrapolation of accumulation curves which had been shown to be similar for BINs and morphotypes^40^. Rarefaction allows the direct comparison of diversity values between differently sampled habitats by calculating an expected number of species based on a fixed sample size. Respective analyses were carried using all trees examined at each elevation and separately for the two tree species *Lithocarpus indutus* and *Sloanea sigun* examined in both forests. In addition, Nonmetric Multi-Dimensional Scaling (NMDS) was computed to analyse differences in the composition of the beetle communities between the two montane forests. Because rare species (singletons) typically raise the level of noise in the data, they are often downweighted or eliminated from analysis. We used the Chord distance as it reduces the weight assigned to rare species^41^. Besides the Chord distance, we did all calculations using the Horn distance due to its relative independence from sample size and diversity, but the results did not differ. For partitioning the resulting distance matrices we applied the Adonis2 approach^42^ as implemented in the vegan package with the following model adjusting also for rarified alpha-diversity of tree species. This approach fits linear models to distance matrices and uses a permutation test with pseudo-F ratios.

Distances = Site + Rarified Diversity + Tree Family + Collecting Sheet + Leaf Cover + Girth at Breast Height. The associated significance test was based on 99,999 permutations.

Throughout the paper, all p-values were adjusted for multiple tests according to Benjamini-Hochberg. We measured beta-diversity for each forest using the Chord distance and tested for significant differences by applying the Adonis2 approach. We also calculated univariate beta-diversity for each tree in a given forest using abundance based Sørensen index^43^. To test if one or more groups was more variable than the others, ANOVA of the distances to group centroids was performed as implemented in the vegan package. Differences in beta-diversity were visualised as boxplots. Furthermore, we focused on the 22 BINs with the highest K2P distance and more than 16 individuals (S-Tab. 3). For these BINs, Tajima’s D^44,45^ with the associated p-value were calculated. Based on the distance matrices for these BINs, significant associations were tested applying the Adonis2 model. Only one BIN showed a significant site association in its haplotype network which was inferred based on the implementation in pegas^44^. For the phylogenetic analysis, all 168 available sequences for this BIN (ADA7144) together with five closely related sequences (BIN ADG2270) were used. The latter BIN served as a root for the inferred phylogenetic tree. Model test as implemented in phangorn^46^ identified the HKY + I model; based on this a maximum likelihood tree a bootstrap consensus tree was inferred.

## Results

### Major taxa composition

**Figure 2:**
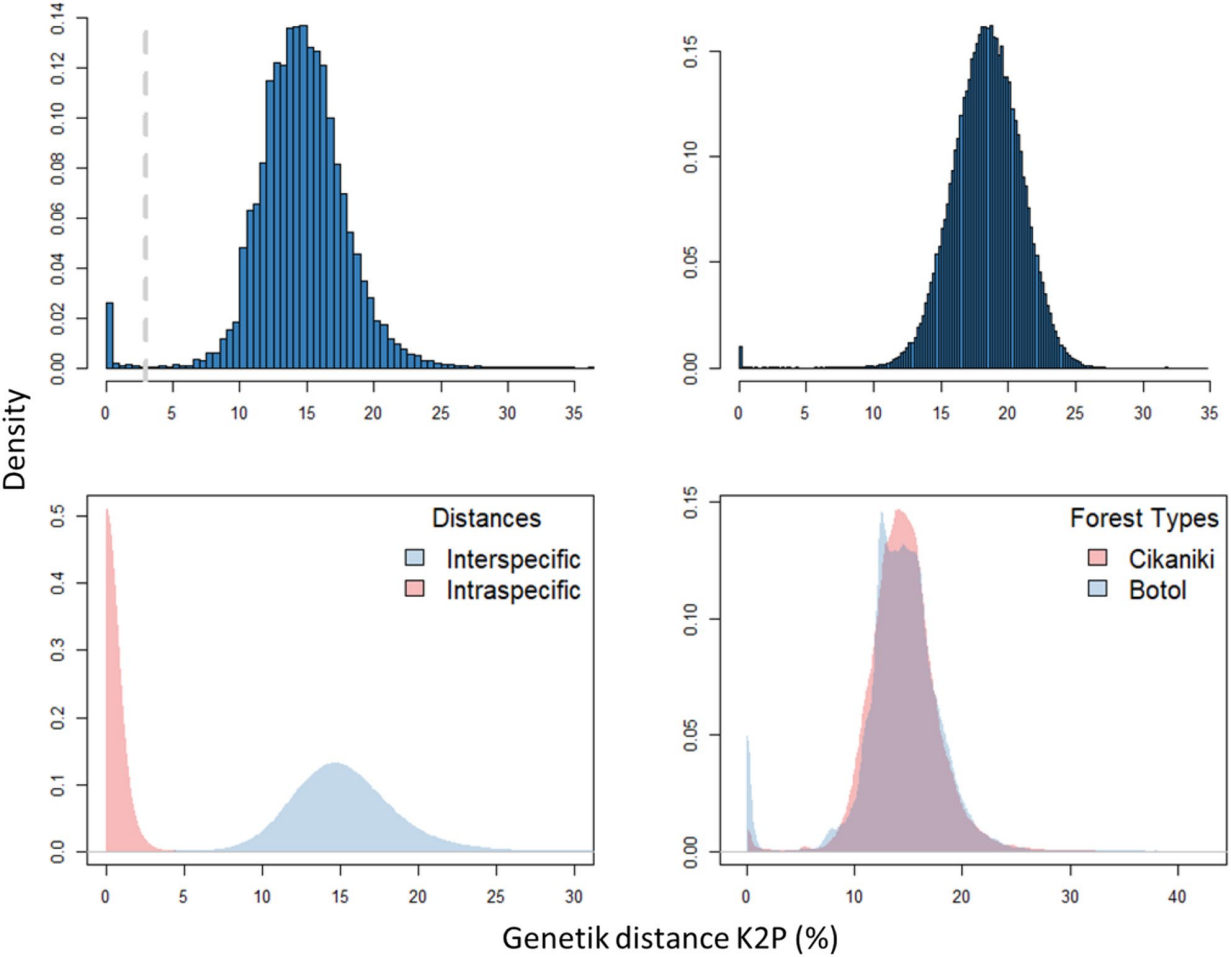
**(A)** Histogram of all pairwise divergences (K2P) for the 3,668 beetle sequences collected in this study showing an average distance of 15%. The dashed grey line indicates a divergence value of 3%. **(B)** The same distribution as in (A) but for 3668 random beetle sequences from BOLD. The left mode represents conspecific beetles while the right mode, which represents interspecific divergences, has a mean value of 18%. **(C)** Based on BIN assignments for the fogged beetles, the maximal genetic distance between intraspecific comparisons (red) and minimal distance between interspecific comparisons (blue) shows a barcode gap indicating a clear break between intra- and interspecific divergences. **(D)** Density plot of genetic distances of sequences for the fogging data set displays significant differences between the lower altitudinal forest (red) and the higher altitudinal forest (blue). The high montane site shows a greater frequency of very similar sequences (Adonis2, p < 0.001).

In total, 43,789 arthropods were collected by fogging (S-Tab. 2). Correspondence Analysis indicated strong differences in the composition of the major taxa between the lower and upper forests (Fig. 1) which can be attributed to differences in the abundance distribution, particularly of the Formicidae and Diptera which were much commoner in the lower forest (S-Fig. 2). Arboreal Formicidae represented a much higher proportion of the community at the low elevation site (32.9% versus 2.0%) while the reverse was true for beetles (12.9% vs. 37.1%) and Opiliones (1.8% vs. 8.2%). Diptera were also found in high numbers and unevenly distributed between forests. These differences in the relative abundance distribution of the major arthropod groups resulted in a clear separation of the two forests in the correspondence analysis (Fig. 1). The first six axes explain 93% of the variation in the data. The number of samples in this analysis differs slightly from the following beetle analysis.

**Figure 3:**
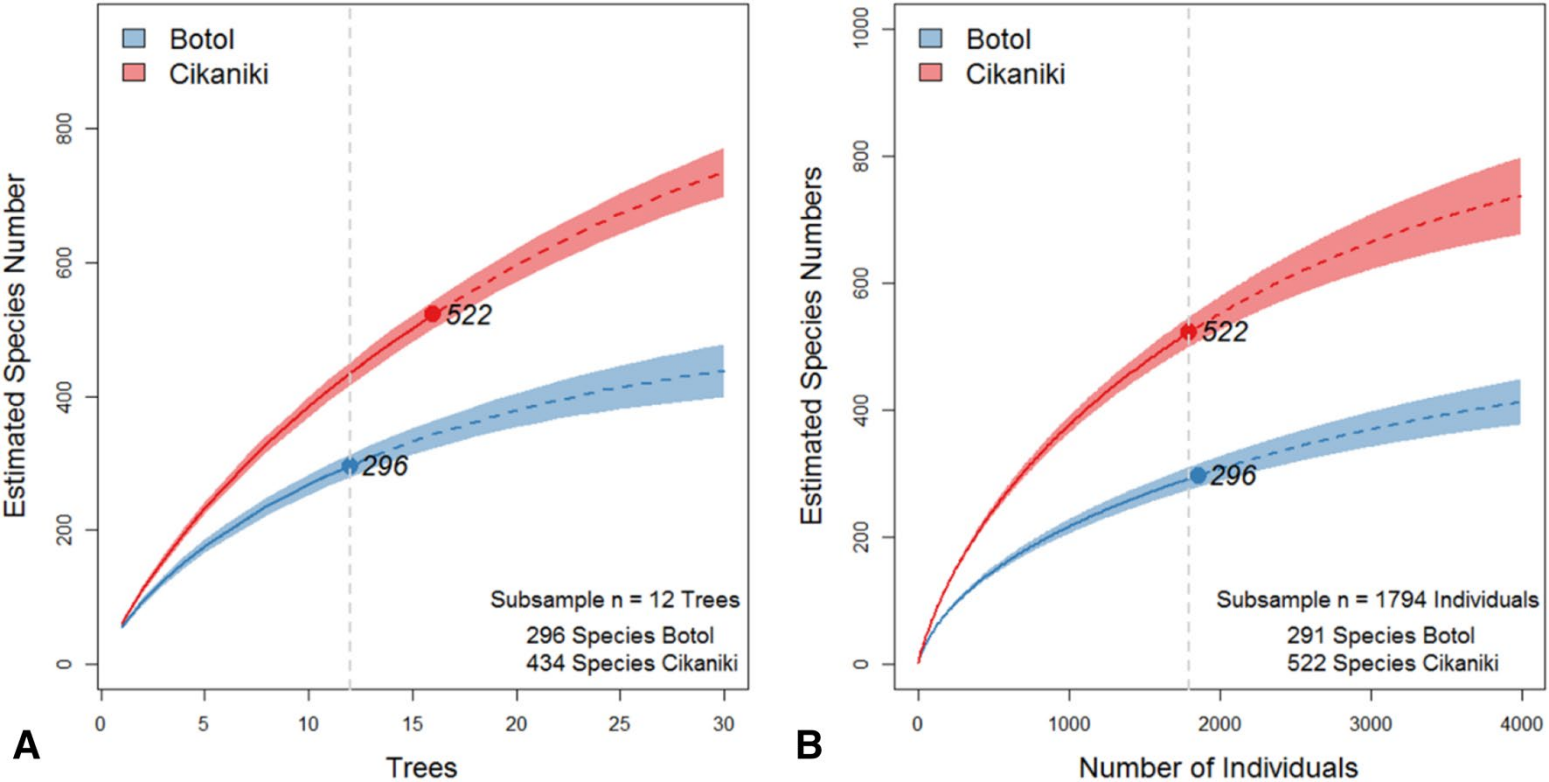
Rarefaction curves with 95% confidence bands calculated for beetle BINs from the lower forest site (Cikaniki) and the higher elevation forest (Botol). Dots represent total number of species (BINs). The grey line marks the differences in species numbers based on a sample of the same number of fogged trees **(A)** and of beetle individuals **(B)**. Exact species numbers encountered in the study are shown.

From the 7543 beetles, sequences were recovered from 3,668 (49%) which represented 752 BINs (Table 1). The number of beetles with sequences but not the number of species was slightly higher at the high elevation site while the proportions of individuals were similar in Botol and Cikaniki (50.9% versus 49.2%). In the lower forest, 522 species were identified; 51.9% were singletons while 296 species were detected in the upper forest of which 48.7% were singletons. Just 33 of the 3,668 sequences representing 11 species showed > 95% identity to existing records on BOLD (S-Fig. 3).

**Table 1:** Number of DNA barcode records and BIN counts for beetles from the low elevational forest (Cikaniki) and the higher elevational forest (Botol) in Mount Halimun-Salak National Park.

Summary statistics	
Number of specimens analysed	7543
Selected sequences > 500 bp	3668
Number of sequences without BIN	20
Total number of BINs	752
Cikaniki	
Sequences (individuals) from Cikaniki	
With BIN annotation	1794
Number of BINs	522
Number of singletons per BIN	271
Botol	
Sequences (individuals) from Botol	
With BIN annotation	1854
Number of BINs	296
Number of singletons per BIN	144

**Figure 4:**
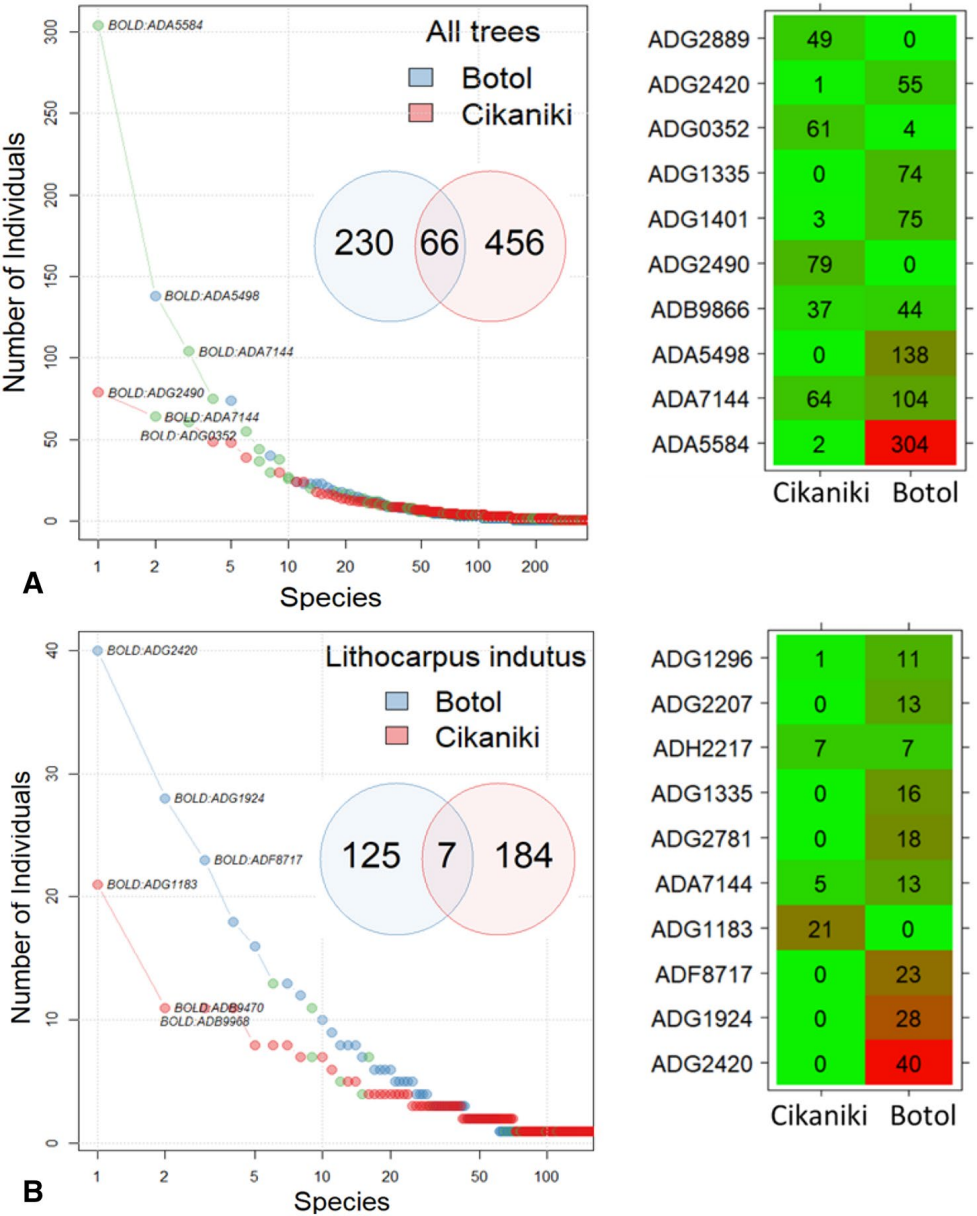
**(A)** Rank-abundance-curves for canopy beetles differed between the lower forest Cikaniki (red) and upper high forest Botol (blue). The green points indicate species occurring at both sites. The inlaid Venn diagram shows the low species overlap between forest sites. The distribution matrix shows the ten most abundant species. **(B) **The same figures but computed only for the *Lithocarpus indutus* trees in Cikaniki and Botol.

Just 66 species are shared between the two sites indicating high beta-diversity (see Fig. 4).

### Divergence of beetle barcode sequences

A histogram of pairwise sequence divergences (K2P) for all individuals (Fig. 2A) revealed very few closely related sequences. Sequences possessed an average K2P divergence of 15% while the highest divergence value was 33%. Furthermore, we compared the distribution of genetic distances in the current dataset with a random subset of 3668 beetle sequences from BOLD (Fig. 2B). The left mode represents the few conspecific beetles while the second mode shows a genetic distance of 0.18. Therefore, this histogram displays a representative picture of beetle divergence in BOLD. The similar patterns of sequence divergence suggest that fogging sampled a similar proportion of beetle diversity from the canopy. Plotting the cumulative sequence similarity for all fogged beetles (this study) to the nearest neighbour in the Coleoptera data from BOLD shows that 99.4% of the fogged species were not previously represented in BOLD. Only 0.9% show less than 5% divergence (S-Fig. 3). The distribution of pairwise genetic distances (K2P) for all intraspecific and interspecific sequences in the beetle sequence data shows a distinct barcode gap at about 3% (Fig. 2C). Figure 2D shows that the beetle communities in the two forests differed in sequence divergence (Adonis2, p < 0.001). The occurrence of abundant species in the higher elevational forest, as indicated by the blue left mode, was conspicuous.

### Ecological characterisation

The BIN assignments were used to generate a community species abundance matrix per tree to estimate expected species numbers. Individual rarefaction curves and sample-based rarefaction curves did not reach an asymptote indicating that many species await collection (Fig. 3). Nevertheless, the curves provide insights into community structure because the steepness of the curves is determined by the abundance distribution of the beetle species. The curves indicate that diversity was significantly higher at the lower elevation site. This conclusion was supported when species richness was compared for the same subsample size as indicated by the vertical grey line. According to this, around 44% fewer species were collected in Botol.

The two forests had largely different beetle communities as only 66 species (8.8%) were collected at both sites (Fig. 4). Species overlap remained low (6.9%) even after excluding singletons or species with more than five individuals (5.3%). Beetles were not evenly distributed between Cikaniki and Botol. The most abundant species were collected at the higher elevation site, but these species were either absent or present in much lower numbers at Cikaniki. The constancy with which species were collected from the trees also differed significantly between forest types and was higher in Botol than in Cikaniki (Wilcox-test, p > 0.0001). The most common species was a member of the subfamily Galerucinae (Chrysomelidae) represented by 304 individuals at the higher site. Species at the lower site were represented on average by 3.5 individuals while the respective number for the high montane site was 5 individuals. The ten most common beetle species comprised 25.7% of all beetles at the lower site but 48.2% at the higher forest site. Species represented by one individual represented 51.9% of all taxa at Cikaniki versus 48.6% at Botol. However, the relative proportion of singletons per tree differed very significantly between the two forests (Wilcox-test, p < 0.0001). The same pattern was found when considering only beetle communities from the tree species *Lithocarpus indutus* and *Sloanea sigun* that was fogged in five trees in Cikaniki and three/two trees in Botol, respectively (S-Fig. 4). The overlap of beetle species between forests was 8.8% for the whole data and 2.2% for the *Lithocarpus* trees.

**Figure 5:**
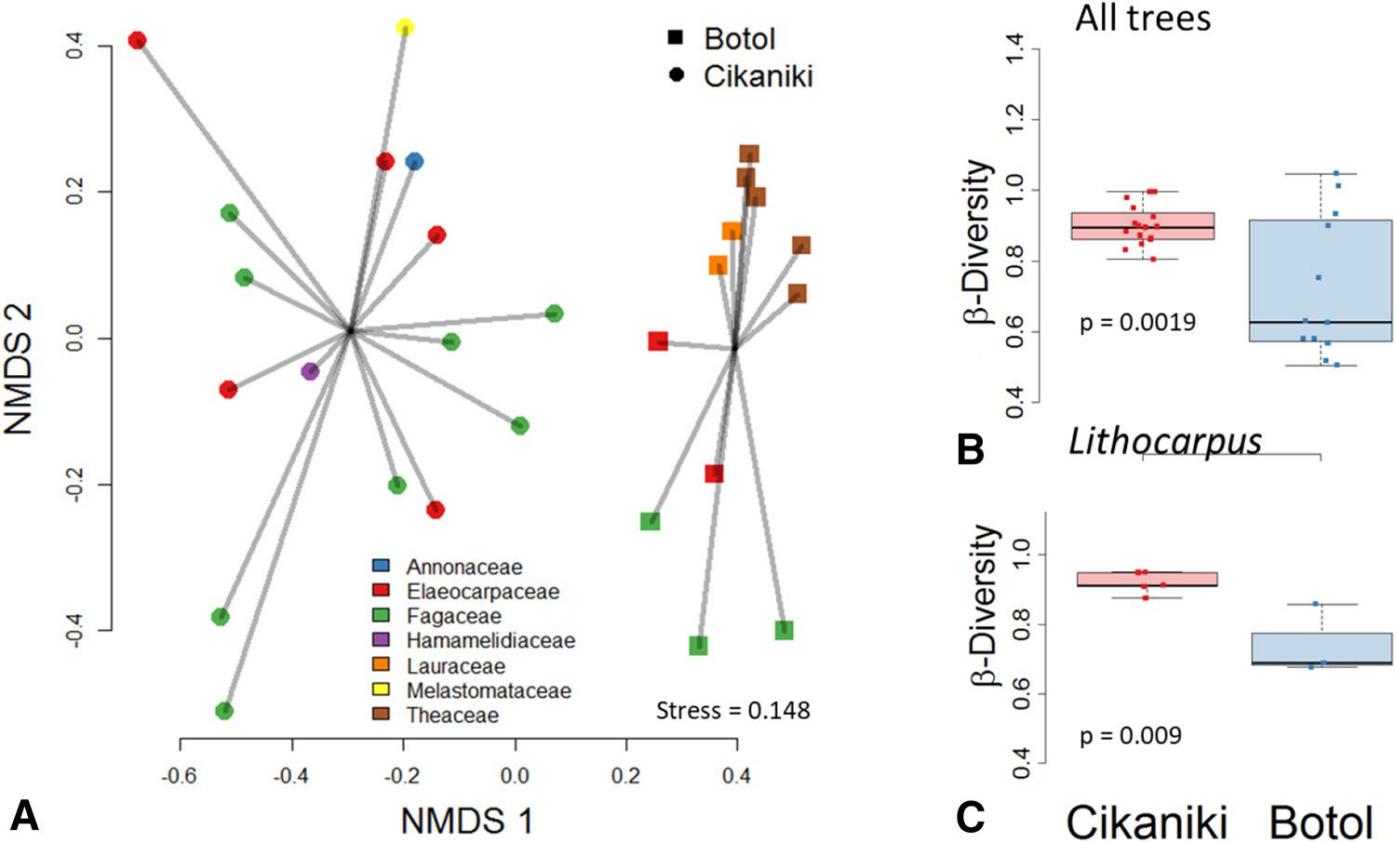
**(A)** NMDS ordination of beetle communities from the canopy of the lower elevational Cikaniki forest (circles) and the higher elevational Botol forest (squares) illustrates differences in compositional similarity which result in full separation. **(B)** Boxplots of beta-diversity differed significantly between Cikaniki and Botol for the whole data as well as **(C)** only for *Lithocarpus* trees.

Beta-diversity between the two forests was high. Besides the low species overlap (Fig. 4), this was evidenced by the clear separation in NMDS ordination (Fig. 5A). Modelling species distributions showed a significant influence of the factors ‘forest site’ and ‘tree family’ (Adonis2, p < 0.001). Rarefied species diversity between trees differed weakly significantly (p = 0.057). Measuring beta-diversity separately for each forest type revealed a significant influence of ‘tree family’ only in the upper forest Botol (Adonis2, p = 0.007). The same results were obtained when calculating the model using the Horn distance measure. Expressing beetle beta-diversity as boxplots of mean distances to centroid illustrate significant differences between both forests (Fig. 5B). Restricting analysis to *Lithocarpus indutus* and *Sloanea sigun* trees respectively, produced similar results (S-Fig. 5).

**Figure 6:**
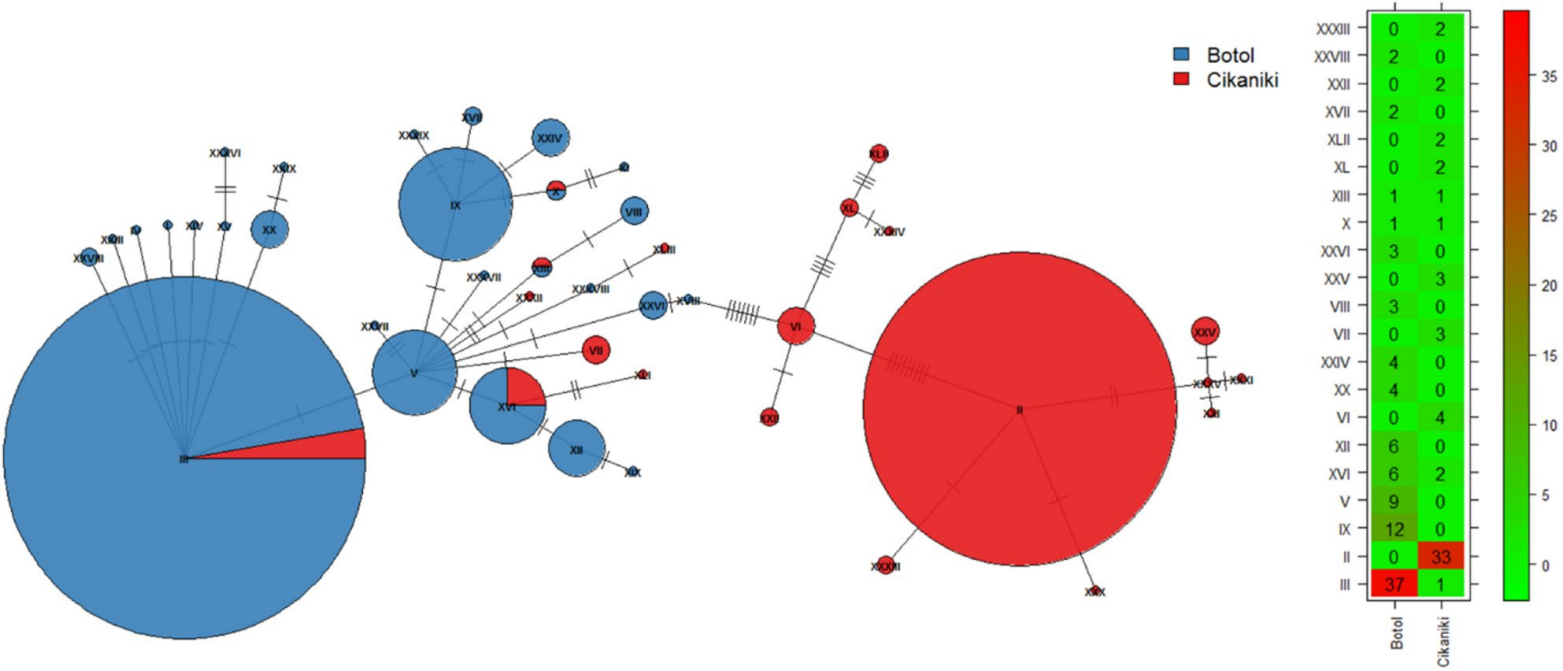
**(A)** The haplotype network for 168 individuals in 43 haplotypes of BIN ADA7144 which was the only BIN with a significant site association. Node size corresponds to haplotype frequency while the colour represents forest. The pies at each node display the distribution of haplotypes. The numbers of nucleotide substitutions between haplotypes are shown by the short lines crossing each connecting line. All haplotypes are annotated by Roman numerals. **(B)** Matrix showing the frequency of haplotypes with more than one individual.

For each of the 22 most abundant BINs (more than 16 individuals, see S-Tab. 3) a genetic distance matrix was modelled with the relevant factors. After multiple corrections, only one BIN showed a significant association between genetic distance and forest sites (BIN ADA7144; Adonis2, p < 0.001). This Ptilodactylidae species (n = 168 individuals) was represented by 104 specimens at Botol and 64 specimens at Cikaniki. Its haplotype network was dominated by two haplotypes [II and III, Fig. 6)]. Although haplotype II was common at Cikaniki, it was not detected at Botol. Specimens from Cikaniki were characterised by sequences which showed very limited sequence divergence while those from the upper forest showed much deeper divergence. Another less frequent haplotype was positioned between the two dominant haplotypes separated by many mutations. Non-neutral sequence evolution was ruled out on all haplotypes (Tajima’s D = − 1.3, not significant; Tab. S-3). However, splitting the dataset into haplotypes from Botol and Cikaniki showed that Tajima’s D was not significant in Cikaniki (D = 0.001, ns) but highly significant in Botol (D = − 2.5, p = 0.001) suggesting a selective sweep. A rooted phylogenetic tree indicates direction of haplotype spread starting from Cikaniki to Botol (Fig. S-6).

## Discussion

The Sunda Islands in SE-Asia are among the most species-rich but threatened ecosystems worldwide^23^. Mountain forests in this region harbour a particularly high proportion of this richness^3^ which is still largely unexplored, especially with regards to life in the canopy^4,5^. Studies from the Kinabalu National Park on Borneo suggest that more than 80% of all beetle species collected from the canopy are new to science^6^. High species richness makes ecosystem studies difficult since differences between species communities often cannot be assigned to particular habitat types (tree species in the present study).

This investigation confirms the high species richness of beetle communities found on trees at both the lower (Cikaniki) and higher (Botol) elevation forests. Documentation of the extent and distribution of canopy diversity is essential to understand the relationship between biodiversity and ecosystem function and to provide the evidence needed to motivate efforts to halt the irreversible destruction of primary tropical forests^47,48^. Our fogging studies revealed clear differences in community diversity and composition between the two elevational forests that were even apparent in the composition of major taxa. This is most evident for the arboreal ants which decrease greatly in abundance and species diversity in the upper forest confirming earlier findings^49,50^. Consequently, the influence of the ants on the canopy fauna should be minimal in high elevations. In lowland rain forests it has been shown that the ants exert a high predation pressure on other arthropods, preventing the establishment of communities with predictable species composition^51^ but see^52^. However, other taxa of great ecological importance, such as the Diptera and parasitic Hymenoptera, need to be analysed to develop a general understanding of the structural components of these ecosystems.

### Beetle diversity

Fogging efficiently collects tree-specific arthropods approximately according to their abundance allowing in-depth analysis of canopy communities. By using DNA barcodes for the discrimination of beetle species^53^, we could also assess the diversity of the beetle community in each forest type and accurately quantify faunal differences. As a consequence of the incomplete knowledge of Indonesia's biodiversity and the expected high number of unknown species^54^ it is unsurprising that 99% of the BINs detected in this study were new to BOLD. Before analysing the data, we tested and found support that the fogged beetles showed similar levels of genetic divergence as a random sample from BOLD. This suggests the subsamples collected by fogging were representative of the investigated ecosystems. The presence of a distinct barcode gap further confirmed that DNA barcodes are an effective tool for species delineation in this setting.

We found that the 1100 m forest (Cikaniki) and the 1700 m forest (Botol) possessed distinct canopy communities. High diversity of canopy beetles in the lower elevational forest and its marked decline in the high forest corresponds with the observation of a mid-elevational peak in diversity in many animal and plant groups^21,55^. Unfortunately, the lowland forests of the Halimun Mountains have already been destroyed for agriculture so a diversity peak at mid-elevations cannot be tested. In addition, diversity maxima show considerable variability among groups of organisms and geographical area^55^. For example, beetles showed a diversity peak at 2100 m a.s.l. in New Guinea^12^ but species numbers of leaf beetles (Chrysomelidae) collected at 1000 and 2000 m a.s.l. in Ecuador were similar^11^. The causes for this variability remain elusive^56^ especially as it is not understood what reduces diversity in lowland forests^49,56^.

For the first time, our results reveal that the transition from the sub-montane to montane forest is associated with fundamental changes in the structure and diversity of insect communities in the canopy. The beetle communities of the lower elevational forest possess high diversity, many rare species, and the compositional dissimilarity between conspecific and heterospecific trees are very similar to results from communities in lowland forests^5,12,17^. By contrast, beetle communities in the mountain forest show a strong dominance hierarchy and greater compositional similarity. These community differences are probably due to greater climatic and seasonal variation typical of higher latitudes^52,57^. Current knowledge suggests that this is due to the increased occurrence of temperate taxa^55^, but a detailed analysis is still pending for the Halimun Mountains. The same results as those for all trees were obtained when analysis focused on two tree species (*Lithocarpus indutus*, *Sloanea sigun*) found at both sites. This supports the argument that these findings are in fact due to forest altitude illustrating the large differences in ecological conditions between the two forest types.

The low incidence of shared beetle species between the two forest is remarkable: Less than 9% of all beetle species were collected at both sites, while the *Lithocarpus* and *Sloanea* trees shared only 2% suggesting that few beetle species can extend their range along the elevational gradient. This result is consistent with the hypothesis that altitudinal dispersal of most species is restricted^21,58^. High turnover rates between insect assemblages along elevational forests were also found in previous studies^11,21,40^ and underline the importance of the elevational forests to overall diversity. Remarkably, different beetle species have adopted the same tree species in each of the elevational forests^28^.

### The distribution of the Ptilodactylidae species along tropical elevational forests

The reason for the strict separation of canopy arthropods could be their narrow thermal tolerances^22^ which increase the costs of dispersal over climatic gradients, resulting in low gene flow and high potential for allopatric speciation^20,21^. Colonizing species must adapt to the new environment to survive. The DNA barcodes of the fogged beetles suggest such adaptation, but such processes are difficult to examine with the present study as few species were abundant in both forests. The present study identified one common species of Ptilodactylidae (BIN ADA7144) which included two sequence subclusters whose distributions differed along the elevational gradient. Tajima’s test statistic and the rooted phylogenetic tree (S-Fig. 5) support the recent unidirectional expansion of beetles from the low to the high montane forest. The large number of haplotypes in this species are perhaps associated with adaptation to the altered habitat conditions but there was no evidence of morphological divergence. This is another example demonstrating the potential of DNA barcodes to reveal potential species complexes consisting of several morphologically similar species with narrow elevational distributions^18,20,59^.

Our data suggest that elevational gradients promote diversification by continuous adaptation of species to changing habitat conditions^60,61^ illustrating the importance of tropical mountain forests for conserving and creating biodiversity. In view of the rapid destruction of these forests^1^, it is critical to employ recent advances in molecular genetics on a large scale to break the taxonomic bottleneck and generate the detailed information on biodiversity at a global scale needed to support sustainable environmental protection.

## Conclusions

This pilot study suggests that communities of adjacent high-altitude forests, separated by only a few hundred metres in elevation, are subject to fundamentally different ecological forces. Diversity assessments ordinarily require comprehensive and tedious taxonomic study. In this study, we combine the efficiency of fogging with the advantage of DNA barcoding to characterise hyper-diverse canopy arthropod communities and to connect biodiversity with population genetics. The high richness of montane forests reflects the proximity of forest strata which separately add to overall diversity. Species colonisation and adaptation result in the juxtaposition of haplotypes eventually fostering speciation. The present results suggest previously unknown fundamental changes in canopy communities in tropical mountain forests, which extend to the genetic level. The generality of these results and whether they apply in other settings will require more extensive investigations examining additional tree species.

## Supplementary information

Supplementary Information.
