# Supplementary material for: Integrative ecological and molecular analysis indicate high diversity and strict elevational separation of canopy beetles in tropical mountain forests
Source: Sci Rep. 2020 Oct 7;10:16677. doi: 10.1038/s41598-020-73519-w (PMC7541450; doi:10.1038/s41598-020-73519-w)
Supplement: Supplementary file 1 — Supplementary Information. [file 41598_2020_73519_MOESM1_ESM.docx]

**Andreas Floren^1,2^, Thomas von Rintelen^3^, Paul D. N. Hebert^4^, Bruno Cancian de Araujo^2^, Stefan Schmidt^2^, Michael Balke^2^, Raden Pramesa Narakusumo^5,6^, Djunijanti Peggie^5^, Rosichon Ubaidillah^5^, Kristina von Rintelen^3^ and Tobias Müller^7^**

**Integrative ecological and molecular analysis indicate high diversity and strict elevational separation of canopy beetles in tropical mountain forests**

**Author affiliations**

^1^ Department of Animal Ecology and Tropical Biology, Biocenter, University of Würzburg, Hans-Martin-Weg 5, D-97074 Würzburg, Germany

^2^ Bavarian State Collection of Zoology, Münchhausenstr. 21, D-81247 Munich, Germany

^3^ Museum für Naturkunde – Leibniz Institute for Evolution and Biodiversity Science, Invalidenstraße 43, D-10115 Berlin, Germany

**^4^**Centre for Biodiversity Genomics, University of Guelph, Guelph, ON N1G 2W1, Canada

^5^ Zoology Division (Museum Zoologicum Bogoriense), Research Center for Biology, Indonesian Institute of Sciences, Jl. Raya Jakarta-Bogor KM 46, Cibinong, Bogor 16911, Indonesia

^6^ Museum of Natural History Karlsruhe, Erbprinzenstr. 13, D-76133 Karlsruhe, Germany

^7^ Department of Bioinformatics, Biocenter, University of Würzburg, Am Hubland, D-97074 Würzburg, Germany

Corresponding author:

Andreas Floren, Department of Animal Ecology and Tropical Biology, University Würzburg, Am Hubland, 97074 Würzburg, Germany, Tel. +49 931 31 84376, Fax +49 931 31 84352,

E-mail: floren@biozentrum.uni-wuerzburg.de

**Supplement**

Supplement Table 1: Number of trees fogged per forest type and tree family.

| **Tree family** | **Tree species** | **Cikaniki** | **Botol** |
| --- | --- | --- | --- |
| Fagaceae | *Lithocarpus indutus* | 5 | 3 |
| Fagaceae | *Castanopsis javanica* | 3 |  |
| Elaeocarpaceae | *Sloanea sigun* | 5 | 2 |
| Annonaceae | *Polyalthia subcordata* | 1 |  |
| Hamamelidiaceae | *Altingia excelsa* | 1 |  |
| Melastomataceae | *Memecylon garcinioides* | 1 |  |
| Theaceae | *Schima walichii* |  | 5 |
| Lauraceae | *Litsea spec.* |  | 2 |

Supplement Table 2: Tree characteristics and number of collected arthropods with standard deviation.

**
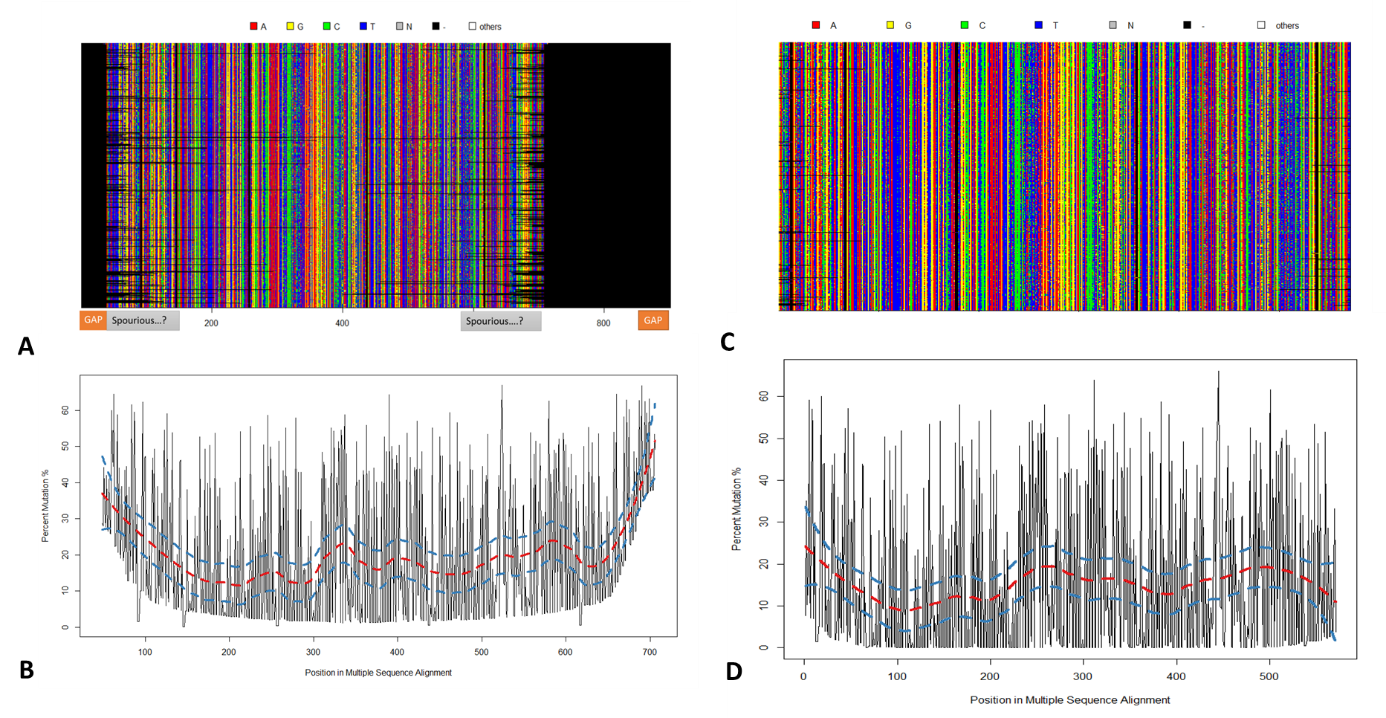
**

Supplement Figure 1: Multiple Sequence alignment before editing (A, B) and after editing (C, D). Start and end regions which contain many gaps and spurious nucleotides were deleted. The lower figures show the percent mismatch relative to the most frequent nucleotide. Blue lines show the 95% confidence bands.

**
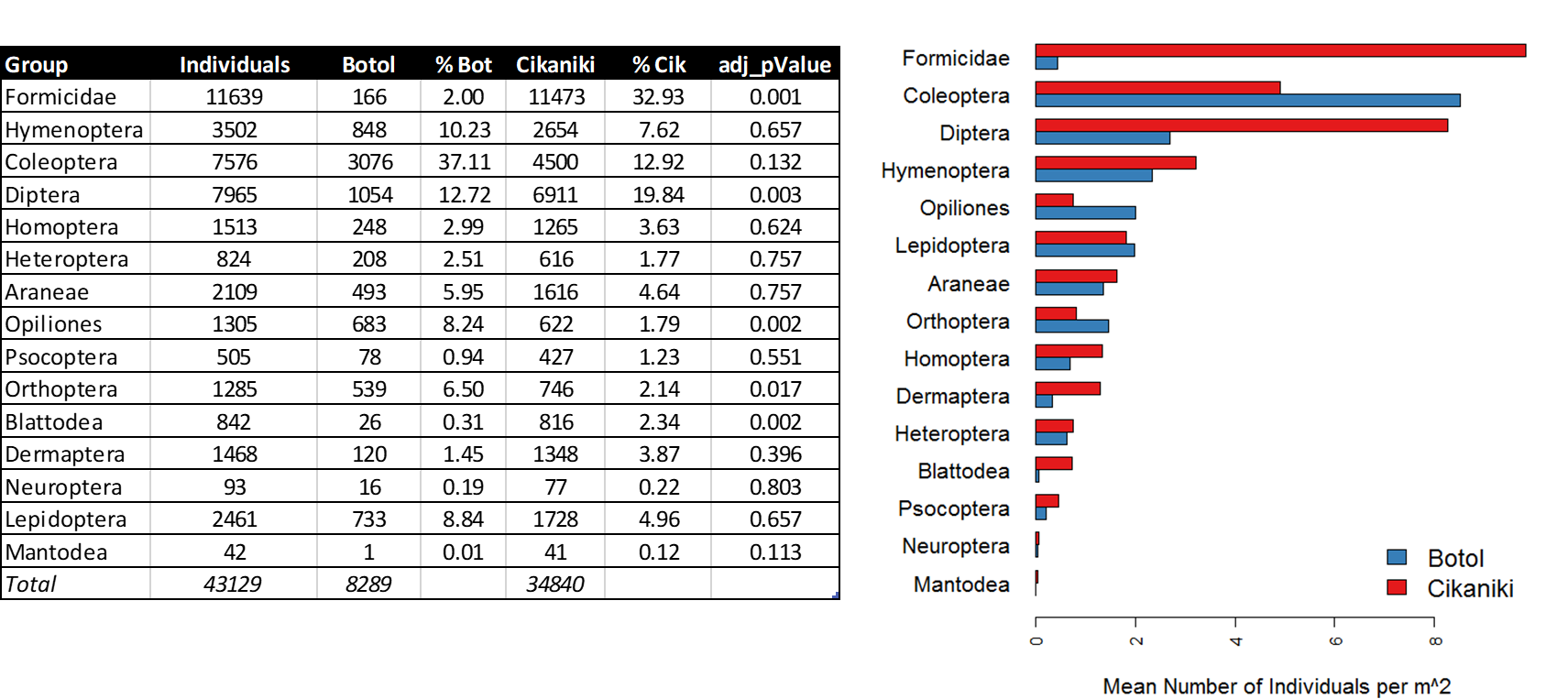
**

Supplement Figure 2: The summary table shows the absolute numbers and percentages of individu-als per major taxon collected from both sites. Mean numbers of individuals per square meter were tested with a Wilcox-test and p-values were adjusted according to the Benjamini Hochberg proce-dure. The barplot illustrates the standardised individual numbers for both Cikaniki (red) and the high elevational forest Botol (blue). Note that the number of beetles in this table is slightly higher than the number given in MS, since a few juveniles were also counted.

**
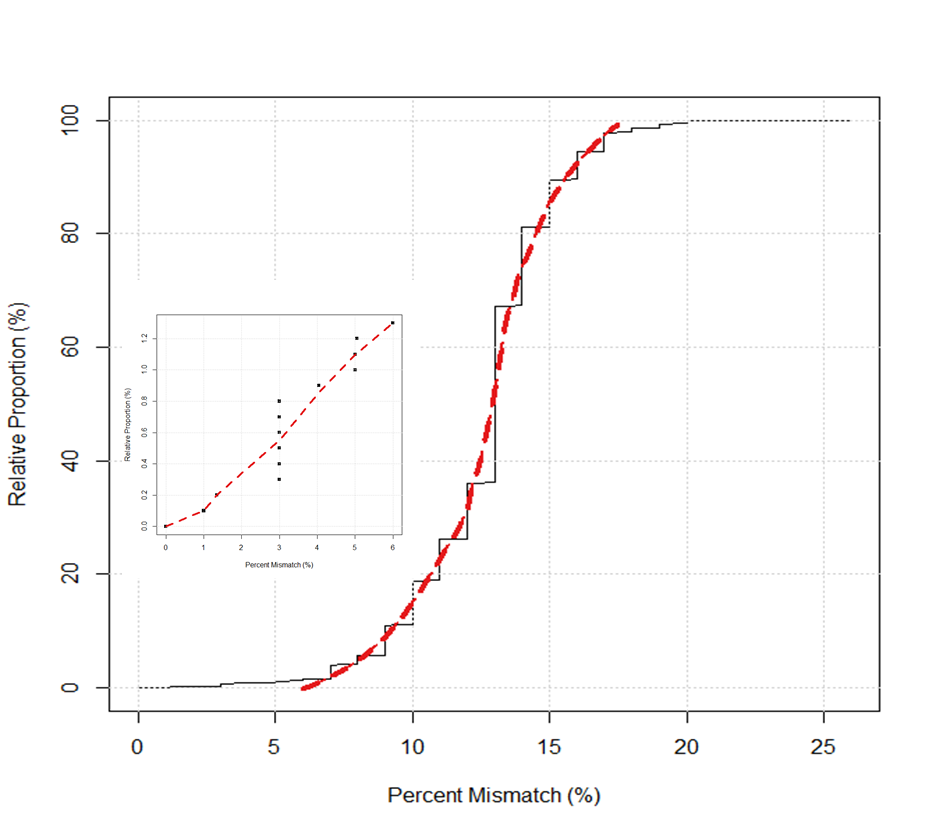
**

Supplement Figure 3: For each of the 3,668 sequences from the fogging samples the best matching sequence of BOLD DB was identified based on BLAST. For each resulting sequence alignment, the percent mismatch was calculated. The cumulative distribution function of all percent mismatches is displayed illustrating the small coverage of the fogged Indonesian beetles in BOLD. Around 1% of the records have less than 5% divergence to the nearest taxon on BOLD. The inlay displays the lower left corner of the figure focussing on 3% divergence.

**
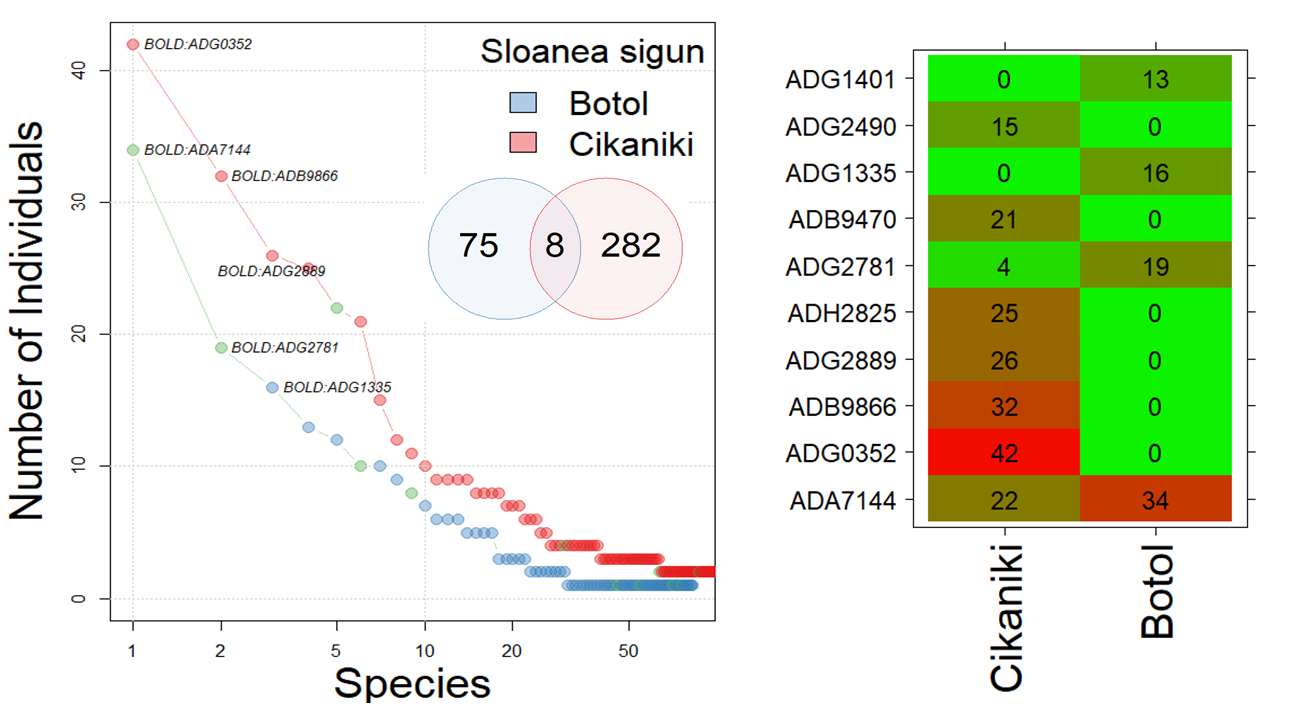
**

Supplement Figure 4: A) Rank-abundance-curves for canopy beetles computed for the tree species Sloanea sigun differed between the lower forest Cikaniki (red) and upper high forest Botol (blue). The green points indicate species occurring at both sites. The inlaid Venn diagram shows the low species overlap between forest sites. The distribution matrix shows the ten most abundant species.

**
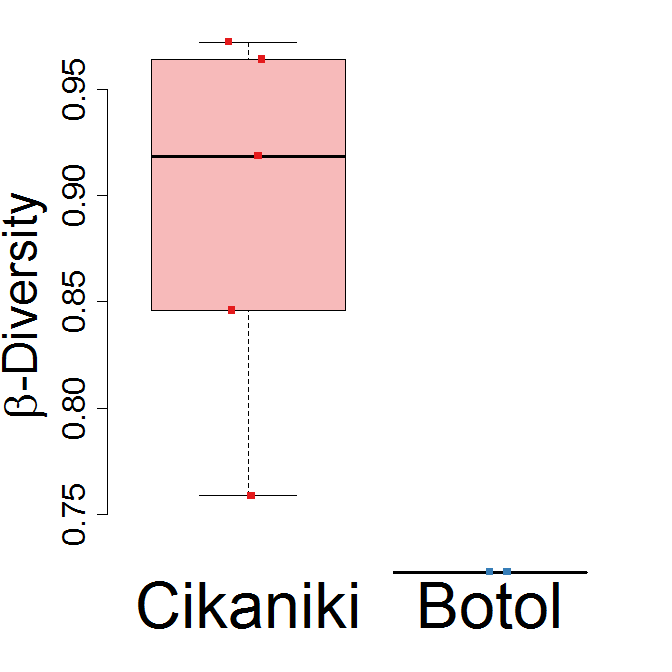
**

Supplement Figure 5: Boxplots of beta-diversity (Chord distance) showed significant differences between Cikaniki and Botol for beetles collected from Sloanea trees. Due to the small sample size (Cikaniki n=5, Botol n=2) differences are borderline significant.

Supplement Table 3: The ten BINs with maximum genetic distances and more than 16 individuals that were used in the analysis. The maximal sequence divergence value, the number of individuals per forest type, and Tajima’s D are shown. Significant values are in bold. Only for BIN ADF 9137 the null hypothesis of neutral evolution was rejected after multiple testing adjustment.

| **BINs** | **K2P Distance (%)** | **Mismatch (%)** | **Individuals** | **Botol** | **Cikaniki** | **Tajima's D** |
| --- | --- | --- | --- | --- | --- | --- |
| BOLD:ADA7144 | 3,22 | 8 | 168 | 101 | 67 | -1,333 |
| BOLD:ADF9137 | 2,4 | 6 | 23 | 23 | 0 | **-3,583** |
| BOLD:ADB9866 | 2,39 | 6 | 81 | 38 | 43 | -1,453 |
| BOLD:ADG1547 | 2,39 | 6 | 17 | 6 | 11 | -1,214 |
| BOLD:ADF8717 | 2,39 | 6 | 23 | 23 | 0 | -0,652 |
| BOLD:ADG2214 | 1,99 | 5 | 23 | 22 | 1 | -2,734 |
| BOLD:ADG2781 | 1,59 | 4 | 48 | 38 | 10 | -0,87 |
| BOLD:ADG1401 | 1,59 | 4 | 78 | 70 | 8 | -2,97 |
| BOLD:ADG0352 | 1,59 | 4 | 65 | 4 | 61 | -2,939 |
| BOLD:ADA9305 | 1,58 | 4 | 28 | 26 | 2 | -2,539 |

**
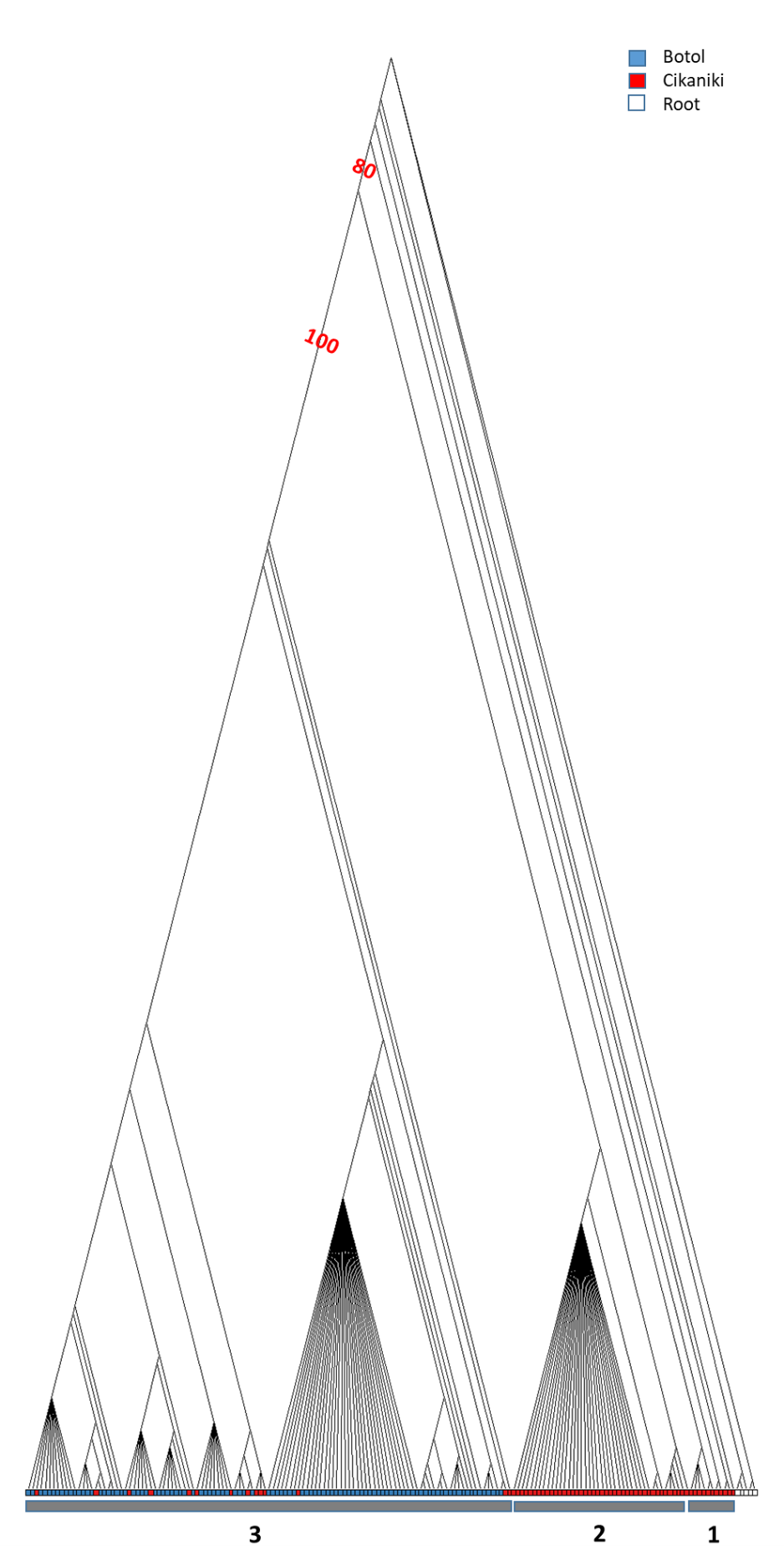
**

Supplement Figure 6: Phylogenetic tree of all sequences for BIN 7144 rooted with sequences of BIN ADG2270 (annotated with white boxes). After root split there are three main splits defining a group of individuals occurring in Cikaniki only with a bootstrap value of 80 (1), suggesting that this species spread from Cikaniki. There are also two sister groups (2) and (3), of which (2) occurs only in Cikaniki, while (3) is intermixed with species mainly from Botol and some from Cikaniki. Both groups are separated by a bootstrap value of 100. Apparently, there are some individuals in group (3) which were rarely observed in Cikaniki.
